# Development and Validation of the Open Matrices Item Bank

**DOI:** 10.3390/jintelligence10030041

**Published:** 2022-07-13

**Authors:** Marco Koch, Frank M. Spinath, Samuel Greiff, Nicolas Becker

**Affiliations:** 1 Individual Differences & Psychodiagnostics, Saarland University, Campus A1 3, D-66123 Saarbrücken, Germany; f.spinath@mx.uni-saarland.de; 2 Department of Behavioral and Cognitive Sciences, University of Luxembourg, 4366 Luxembourg, Luxembourg; samuel.greiff@uni.lu; 3 Individual Differences & Psychodiagnostics, Greifswald University, Franz-Mehring-Str. 57, D-17489 Greifswald, Germany; nicolas.becker@uni-greifswald.de

**Keywords:** intelligence, computer-based testing, item banking, figural matrices, test equating, test development

## Abstract

Figural matrices tasks are one of the most prominent item formats used in intelligence tests, and their relevance for the assessment of cognitive abilities is unquestionable. However, despite endeavors of the open science movement to make scientific research accessible on all levels, there is a lack of royalty-free figural matrices tests. The Open Matrices Item Bank (OMIB) closes this gap by providing free and unlimited access (GPLv3 license) to a large set of empirically validated figural matrices items. We developed a set of 220 figural matrices based on well-established construction principles commonly used in matrices tests and administered them to a sample of N = 2572 applicants to medical schools. The results of item response models and reliability analyses demonstrate the excellent psychometric properties of the items. In the discussion, we elucidate how researchers can already use the OMIB to gain access to high-quality matrices tests for their studies. Furthermore, we provide perspectives for features that could additionally improve the utility of the OMIB.

## 1. Introduction

Figural matrices tasks represent a well-established class of tasks in intelligence tests that load highly on general intelligence (Carpenter et al. 1990; Jensen 1998; Marshalek et al. 1983) or, more specifically, on fluid reasoning (Gignac 2015) and are part of many broad intelligence test batteries (e.g., Wechsler 2008). Fluid reasoning is an integral part of modern intelligence models (Carroll 1993; McGrew 2005, 2009) and has a decisive role in many aspects of a human’s life, such as occupational success (Schmidt and Hunter 2016), educational attainment (Roth et al. 2015), and health (Gottfredson and Deary 2004). Thus, figural matrices tasks are a popular and powerful instrument to answer many practical and scientific questions. In the context of psychodiagnostics, to our knowledge, most established measurement instruments are offered on a commercial basis only. Although noncommercial tests might be of equal psychometric quality, commercially distributed tests are usually better documented and easier to compare. As is the case for raw research data, it is therefore desirable that the means to gather those data are also openly available, which can serve as an accelerator to science (Woelfle et al. 2011). The current study therefore aims to introduce a database of 220 figural matrices items with a broad range of item difficulties that is entirely free and fully accessible for scientific use.

### 1.1. Figural Matrices

Traditional figural matrices tasks implement a distractor-based response format in which the testee is presented with a fixed number of response options from which one option must be selected. Test-takers must inspect the item stem and extract the underlying rules by means of inductive reasoning. Once the rules have been identified, test-takers must envision the correct response and select it from the response options provided. However, it has been shown that this approach results in some amount of uncertainty about to which degree participants actually engage in inductive reasoning and to which degree they rely on response elimination strategies (Carpenter et al. 1990).

An alternative to this approach has been proposed by Becker and Spinath (2014), who developed and validated a construction-based figural matrices task (DESIGMA). There is evidence that this approach improves the construct validity of figural matrices (Arendasy and Sommer 2013; Becker et al. 2016). Furthermore, the DESIGMA items’ construction can be described with simple mathematical and logical operations (Becker et al. 2016; Becker and Spinath 2014) that provide a framework to classify the resulting items. Moreover, the test developers have provided evidence that the strongest known predictor of item difficulty is the number of construction rules underlying an item (r = −.49). Nonetheless, a large amount of variance in item difficulties is unaccounted for. In another study, further information (e.g., perceptual organization, amount of information) was used as a predictor, which resulted in 87% explained variance (Primi 2014).

Despite this advantage, to our knowledge, the DESIGMA (Becker and Spinath 2014) is the only publicly available construction-based figural matrices test. Unfortunately, it is not free for scientific use. While the commercial distribution by publishers does have advantages, such as protection of the test material, we believe that researchers should have access to state-of-the-art measurement instruments free of charge. The item bank that we introduce and evaluate in this article was mainly developed for scientific use and consists of over 200 items with a construction-based response format, making it unlikely that testees are motivated or even able to memorize all solutions. Furthermore, the results presented by Levacher et al. (2021) indicate that learning the underlying rules will not impair construct validity.

### 1.2. Item Banks

Item banks are a collection of test items aimed at a specific construct (Chituc et al. 2019; Ward and Murray-Ward 1994) and are usually built on items tested under the assumptions of IRT models (Bjorner et al. 2007). This has the advantage that all items measure the same construct on the same scale and can therefore be combined in arbitrary combinations (Weiss 2013), which enables researchers to tailor a test that exactly fits their needs (e.g., short test duration, only very difficult items, a special subset of characteristics, etc.) without impairing the resulting test’s item properties. Furthermore, especially for longitudinal studies or studies with repeated measures designs, it is essential that participants be presented with different items for each measurement time. While some traditional figural matrices may consist of two parallel forms, this is very rare to our knowledge. Moreover, the items from an IRT-based item bank can be used for adaptive testing, allowing for more sophisticated assessment procedures.

One common challenge for the development of item banks is that a large number of items need to be included and analyzed regarding their psychometric properties. Oftentimes it is impossible to administer all items to a single sample to avoid systematic measurement error (e.g., fatigue, dwindling motivation). Therefore, items are usually spread across several subsamples. Although this approach could introduce some amount of bias due to random differences between the subsamples, this problem can be mitigated by statistical methods. Anchor-based test equating describes a method that uses shared items (anchor items) between test forms to estimate a transformation coefficient (Battauz 2017). This coefficient can then be used for a linear transformation of the unique items per test form. Consequently, all items are expressed on the same measurement scale, an important prerequisite for a useful item bank.

### 1.3. Aims of the Present Study

With the above in mind, the current study has four goals:

1. develop 220 items that are based on the construction principles described by Becker and colleagues (Becker et al. 2016);
2. evaluate the psychometric properties of all items (e.g., item difficulties and part–whole correlations from classical test theory);
3. test the items under the assumption of IRT models and eliminate the nonfitting items;
4. provide a dataset of the resulting item bank with detailed information on each item.

## 2. Materials and Methods

### 2.1. Sample

A total of 4657 applicants to medical schools in Germany registered for the current study. After excluding 1076 participants who skipped the figural matrices and a further 1020 participants who gave no responses, the final sample consisted of N = 2561 participants, of whom 1870 participants (73.02%) identified as female, 643 participants (25.11%) identified as male, 3 participants identified as nonbinary, and 45 participants (0.02%) did not submit their gender. On average, participants were 19.34 years old (SD = 2.58; range = 15–45).

### 2.2. Procedure

The figural matrices test was one subtest of a test preparation study consisting of several tests of cognitive abilities and natural sciences. The order in which tests were administered was randomized (therefore, participants interested in only certain tasks sometimes skipped whole subtests). The complete preparation study was administered in a self-paced and unproctored online study allowing participants to take breaks between each subtest but not within items of a subtest. Upon starting the figural matrices test, participants were presented with instructions on how the task was to be solved and were then required to solve two practice items. For the two practice items, feedback was given on whether the response was correct and what was wrong in case of a mistake. When the practice items were finished, participants were allowed to work on 28 figural matrices tasks without a time limit. For these items, no feedback regarding the response was given.

### 2.3. Development of the Figural Matrices Items

For the item bank, we chose to use the six construction rules described by Becker et al. (2016) to develop a total of 220 items: (1) addition—elements from the first and second cell in a row are added; (2) subtraction—elements in the second cell are removed from the elements in the first cell; (3) disjunctive union (single element addition)—elements that appear simultaneously in both the first and second cell are eliminated; (4) intersection—elements that do not appear in both the first and second cell are eliminated; (5) rotation—elements rotate (counter-)clockwise throughout the row; and (6) completeness—in every row a certain set of symbols must be represented. The rules have been applied to simple geometric shapes that have been selected for distinctiveness in order not to confound inductive reasoning with individual differences in visual perception.

To ensure that the resulting item bank could be used for testing in every ability range, item construction was based on a normal distribution of rules (e.g., many items with three rules and fewer items with five rules), which can be seen in Table 1. Furthermore, Table 1 represents how often each construction rule was used (for a detailed overview of which combinations of rules were used in each item, please refer to Table S1). Two students were each provided with a list of items and constructed them with a tool developed by the first author. For each item, an algorithm tested whether the item was solvable and whether the demanded rules were implemented.

The items were divided into 10 test sets of 22 items each (Table S1). Each test set consists of two items with one rule, five items with two rules, eight items with three rules, five items with four rules, and two items with five rules. Furthermore, because it could not be guaranteed that each test set consists of perfectly comparable items and because each participant would only take one test set, six anchor items were developed and added to each test set. Two anchor items had three rules; for all other rule counts, only one anchor was used. This approach has been developed and validated in a recent simulation study (Weber 2021).

**Figure 1:**
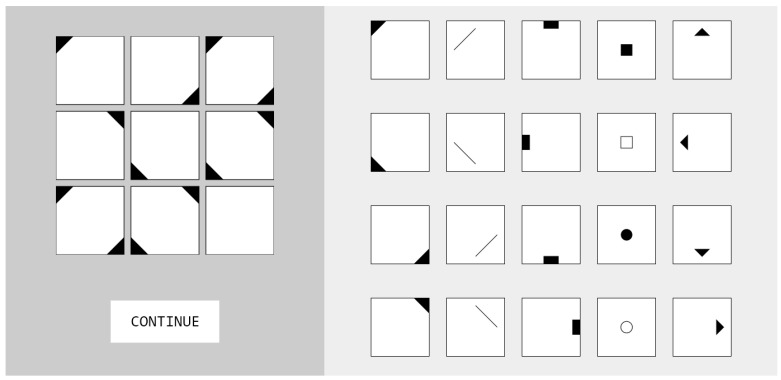
Example item from the current study. On the left, the item stem is presented with eight cells being filled and the last one left empty. Participants are required to use the construction elements on the right to construct the correct response. In this item, the corner elements are added within the rows; thus, the correct response would be to select all four corner elements. Clicking on the button would switch to the next item until all items were solved.

All items were exported as vector graphics, and the test environment was deployed with Unipark Questback EFS (unipark.com). Participants were presented with the item stem on top (a 3 × 3 matrix with the last cell left empty) and the 20 construction elements needed for construction of the response below (Figure 1). Clicking a construction element once would highlight it with a red border; a second click would deselect the element.

### 2.4. Statistical Analyses

Unless stated differently, all statistical analyses were carried out with the statistics software R (R Core Team 2021), and the alpha cutoff for significance testing was set to *α* = .05.

To analyze item parameters, we calculated item difficulty and part–whole correlations in the sense of classical test theory for each test set of figural matrices items separately with the R package *psych* (Revelle 2021). To identify misfitting items, each test set of figural matrices was analyzed with the R package *mirt* (Chalmers 2012), and the cutoffs proposed by Wilson (2005) were applied (i.e., combination of infit or outfit < 0.75, or infit or outfit > 1.33, and significant *t*-statistic). For all items fitting the 2 PL model, we estimated item threshold (*b*) and item discrimination (*a*) with the *equateMultiple* R package (Battauz 2021), which allows for the usage of anchor items in order to control for differences in the samples.

To test that the items were all loading on a single factor as in the original DESIGMA (Becker and Spinath 2014), a multigroup CFA (MGCFA) was calculated with the item test sets as grouping factor. For this, the R package *lavaan* (Rosseel 2012) was used. Three levels of measurement invariance were tested: (1) configural CFA where only the structure is fixed, (2) strong invariance with loadings and intercepts being fixed, and (3) strict invariance with additionally residual variances being fixed (Schroeders and Wilhelm 2011). Due to the dichotomous nature of the data, the WLSMV estimator was used. The model was assumed to be well-fitting if RMSEA was smaller than 0.06, SRMR was smaller than 0.08, and CFI was bigger than .95 (Hu and Bentler 1999).

## 3. Results

**Figure 2:**
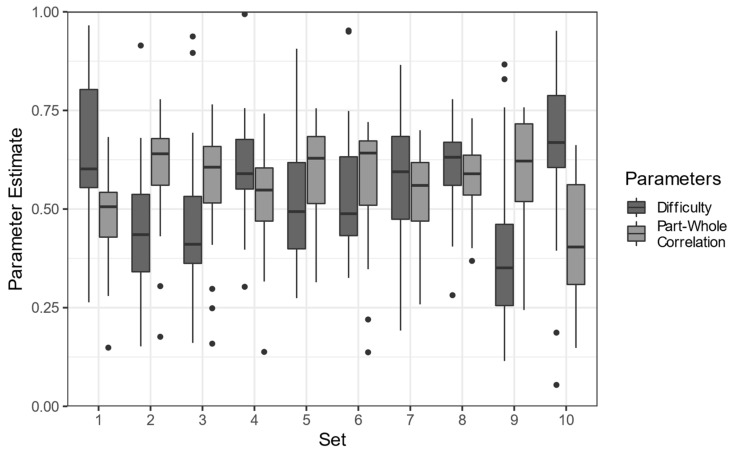
Item difficulties and part–whole correlations as estimated by classical test theory. Black dots represent outliers.

Out of the 28 items per test set, participants solved on average 16.82 items (SD = 7.79), with only n = 25 participants (1%) solving all items correctly. Participants spent on average 26.05 min (SD = 7.07) on the set of tasks. Overall, the developed items were of medium difficulty (M = .55, SD = 0.19) and medium part–whole correlations (M = .54, SD = 0.19). The 10 test sets differed significantly in their difficulty (*F*_(9,210)_ = 5.33, *p* < .001, *ω^2^* = .15) and part–whole correlations (*F*_(9,210)_ = 5.33, *p* < .001, *ω^2^* = .15) as depicted in Figure 2. Internal consistency was high for all test sets (*α* = .92, SD = .02).

One item was identified that did not fit the 2 PL model (outfit_(Item 7)_ < 0.001, *p* < .001) and was excluded from the estimation of item difficulty and discrimination parameters. The average item difficulty parameter after test equating was *M_b_* = -0.17 (SD*_b_* = 0.99), and the average item discrimination parameter was *M_a_* = 2.09 (SD*_a_* = .84). Table 2 depicts the distribution of item parameters depending on the number of rules employed per item (for single item estimates, refer to Supplementary Table S1). The test equating was successful, with no significant differences between the subtests (item discrimination: *F*_(9,209)_ = 1.42, *p* = .180, *ω^2^* = .02; item difficulty: *F*_(9,209)_ = 0.98, *p* = .458, *ω^2^* < .01). The solution probability of each item and the item difficulty as estimated by the 2 PL model correlated highly (*r* = −.68, *p* < .001) and rose substantially after correcting for extreme outliers (i.e., ±3SD; *r* = −.83, *p* < .001). Furthermore, item difficulty was strongly correlated to the number of rules underlying an item (*r* = .53, *p* < .001). In a regression with all six unique rules as predictors, 34% of the variance in item difficulties were explained, *F*_(6,218)_ = 20.31, *p* < .001. The increase in explained variance in comparison to the number of rules was statistically significant, *F* = 5.34, *p* < .001. Except for completeness, all regression weights were significant (Table 3).

The configural measurement invariance model had overall good model fit; however, the SRMR exceeded the predefined cutoff value (CFI = .991, RMSEA = .047, SRMR = .097). The strong measurement invariance model (CFI = .969, RMSEA = .084, SRMR = .138) fitted significantly worse than the configural model (Δ*Χ*^2^ = 448.96, Δ*df* = 234, *p* < .001, ΔCFI = .22). Due to the lack of fit for the strong measurement invariance model, the strict model was omitted.

## 4. Discussion

The purpose of this study was to develop an item bank of figural matrices that is free for scientific use and can be used in many diagnostic settings. To this extent, 220 original figural matrices tasks were constructed, and their psychometric properties were evaluated in a large field study. All but one item fit well under the assumptions of a 2 PL model, suggesting that the construction of a homogeneous figural matrices test was successful. Because it was not possible to administer all 220 items to all participants, a test equating approach was employed in which 10 test sets of items were created and supplemented with six anchor items. The significant results of two ANOVAs comparing the subtests regarding the item difficulties (in the sense of classical test theory) and part–whole correlation underlined the necessity of such an approach.

It was assumed that the item construction method for this item bank would result in a large pool of items with medium item difficulty and few items with very high or low difficulty levels. On average, the items were solved by 55% of the participants. This notion is further corroborated by the average IRT item difficulty parameter *b* = −0.17. This indicates that the developed items were somewhat easier than expected. Nonetheless, as only 25 participants managed to solve all items, no ceiling effects need to be considered. This, in combination with the average item discrimination parameter *a* = 2.09, can be considered evidence that the figural matrices items in this study are appropriate for differentiating between various ability levels.

In accordance with prior studies, there was also evidence for the number of rules being used in constructing an item to be a strong predictor of item difficulty (Becker and Spinath 2014); however, analysis with all rules as predictors explained even more variance. This finding is highly relevant for the further development and improvement of the Open Matrices Item Bank and figural matrices tasks in general, as it allows for more precise development of specific item difficulties if needed. In addition, to a certain degree, the association between construction principles and item difficulty can help to disentangle the association of item and sample parameters. Furthermore, it has to be noted that the approach presented by Primi (2014) explained a larger share of variance. Therefore, further studies should take a more experimental approach to varying item properties in order to gain further insight into the determinants of item difficulties.

Further, in an MGCFA, it could be shown that while the factor structure was invariant throughout all 10 test sets of items, there was no evidence for strong measurement invariance (i.e., identical thresholds and loadings). This was congruent to the assumption that all tests should measure the same construct (i.e., fluid reasoning) but that raw mean values could not be compared without the use of test equating procedures. Accordingly, there was also evidence that the solution probabilities or item difficulties as described by classical test theory were significantly different between all 10 item sets, whereas those differences disappeared in the IRT- and test equating-based item difficulties. This further emphasizes that in large-scale test settings, to select or deselect individuals from a large pool of test-takers, they should all be administered the same items. If that is impossible, test equating strategies such as the use of anchor items (Battauz 2021) must be employed.

There are, nonetheless, three potential limitations concerning the present study. The first limitation is associated with the selected sample. To lay a foundation for the Open Matrices Item Bank, the development of a large item pool was central. Accordingly, a large sample was needed, and the test preparation study for medical school admission tests was a fitting opportunity to validate the figural matrices tasks. Nonetheless, even though this was no student sample, some degree of preselection cannot be ruled out. Until recently, a high GPA was nearly mandatory to be admitted to medical schools in Germany; this might cause many potential applicants to not take part in the costly admission tests (they are associated with costs of 100€) because they doubt their chances at success. However, the fees can be waived if they pose an unreasonable challenge to potential applicants. Furthermore, the goal of the student admission tests for medical universities is to enable applicants with school grades that would prevent them from studying medicine. Therefore, the actual extent of preselection cannot be estimated accurately. Furthermore, it is reasonable to assume that a sample consisting of applicants for medical schools is restricted in the variance of g. This could have an impact on the estimation of item difficulties resulting in an underestimation of real item difficulties. Consequently, this could also impair the predictability of item difficulty by the underlying construction rules. Further, the sample was skewed toward female applicants, which, in turn, might have affected parameter estimates, as there is evidence of an advantage of male test-takers in these types of tasks (Waschl and Burns 2020). While the variance restriction might not be as strong as in a student sample (i.e., only those applicants who have been admitted), further studies should inspect item parameters with a more heterogeneous sample. A second potential limitation is the lack of a time limit and no proctoring for the figural matrices tasks, which might also partly explain why the item difficulties were easier than expected. It has also been shown that unproctored testing is associated with significantly higher test scores (Steger et al. 2018); however, due to the COVID-19-related contact and travel restrictions, traditional group tests were not feasible. The third limitation of the study is the scarce availability of validity data. While the results of the MGCFA lay a strong foundation for the construct validity of the OMIB data, at present, no data for convergent, discriminant, or predictive validity are available. However, as the participants of this study also agreed to the collection and scientific use of their academic achievements (e.g., grades), these data will become available in the future, once the majority of participants have reached certain milestones in their studies.

Despite these limitations, the current data suggest that the figural matrices of the OMIB can be used in unproctored settings as well since only 1% of applicants were able to solve all items. While the setting of the current study was no high-stakes situation, the possibility to prepare themselves for the real admission test should have motivated participants to do their best to fully understand the tasks and improve their chances at admission.

The current article has provided a strong foundation for the OMIB; however, its development is far from finished. Not only is it desirable to increase the current item pool so that the reuse of items becomes virtually unnecessary, but there are also further avenues for research that would deepen the understanding of figural matrices tasks and fluid reasoning. For example, the current construction strategy and documentation of constructed items would allow for the development of parallel tasks in which the only difference would be the used symbols (e.g., round shapes instead of pointy ones or different shadings), which could then be used to deepen our understanding of how visual complexity is associated with the difficulty of figural matrices tasks. Furthermore, in the current study, participants had to construct the response in working memory, and an empty cell would indicate no change when selecting response options. Prior studies have shown that performance in figural matrices is strongly associated with working memory (Kyllonen and Christal 1990; Wilhelm et al. 2013) and that this might be due to the filtering of relevant features (Krieger et al. 2019). If the last cell could indicate which options a test-taker has clicked, this should alleviate some strain on the working memory and improve overall performance. In contrast, the last cell could also be filled with all construction elements requiring participants to delete the incorrect ones. This might increase the demand on filtering processes and impair overall performance.

The present study contributes to a large body of literature regarding the importance of fluid reasoning and primarily its assessment. A total of 220 figural matrices tasks were developed and tested in a heterogeneous sample to provide detailed estimates regarding their psychometric properties. They were of moderate difficulty with some very difficult and some very easy items, allowing for the administration in various diagnostic settings. All items, the rules used to construct them, as well as their properties are provided in the Supplementary Materials and can lay the foundation for open diagnostics in parallel with the ongoing open science movement.

## 5. Access to the OMIB

All information needed to implement the OMIB into research projects can be found on OSF (https://osf.io/4km79/) in the Supplementary Materials. The repository contains a template for pen-and-paper tests, image files of all item stems, a table that contains all item properties (e.g., 2-PL parameters, rules used), and an example code for its distribution as a computer-based test on survey sites. These files are accompanied by an instruction that summarizes how the OMIB can be used, what variables can be customized, and how the resulting test is to be scored.

## Figures and Tables

**Table 1:** Properties of the developed items.

Rules	Number of Items	Add	Sub	Dis	Int	Rot	Com
One	20	4	4	3	3	3	3
Two	50	20	16	16	16	16	16
Three	80	44	44	37	41	37	37
Four	50	35	36	32	35	31	31
Five	20	17	17	17	17	16	16
Sum	220	120	117	105	112	103	103

Notes: Add, addition; Sub, subtraction; Dis, disjunctive union; Int, intersection; Rot, rotation; Com, completeness.

**Table 2:** Item parameter estimates per rule combination.

Rules	*a*	Min*_a_*	Max*_a_*	*b*	Min*_b_*	Max*_b_*
One	1.45	0.11	3.16	−1.87	−8.98	1.43
Two	1.52	0.62	2.97	−0.30	−2.25	1.44
Three	2.01	1.05	3.63	−0.16	−1.12	1.65
Four	2.64	1.08	5.16	0.24	−0.12	0.88
Five	3.10	1.63	4.48	0.67	0.27	2.41
Average	2.09	0.11	5.16	−0.17	−8.98	2.41

Note: *a*, item discrimination parameter; *b*, item difficulty parameter; Min, minimum; Max, maximum.

**Table 3:** Item difficulty prediction.

Rules	*B*	*β*	*t*	*p*
Intercept	−1.56	---	---	---
Addition	0.41	0.21	3.84	<.001
Subtraction	0.51	0.26	4.84	<.001
Disjunctive union	0.77	0.40	7.20	<.001
Intersection	0.68	0.35	6.43	<.001
Rotation	0.34	0.17	3.16	.002
Completeness	0.12	0.06	1.11	.267

Note: *B*, regression weight; *β*, standardized regression weight. The test statistic for the intercept was omitted, as it holds no meaning (i.e., items without at least one rule do not exist).

## Data Availability

The datasets presented in this study can be found in online repositories.

## Supplementary Materials

The following are available online at https://www.mdpi.com/article/10.3390/jintelligence10030041/s1, Table S1: construction rules and item characteristics. Vector graphics of all items and test templates are available online at https://osf.io/4km79/.
