# Supplementary material for: Development and Validation of the Open Matrices Item Bank
Source: J Intell. 2022 Jul 13;10(3):41. doi: 10.3390/jintelligence10030041 (PMC9326670; doi:10.3390/jintelligence10030041)
Supplement: Supplementary file 1 [file jintelligence-10-00041-s001.zip › jintelligence-1770384-supplementary.pdf]

Journal of Intelligence

MDPI

Table S1. Construction rules and item characteristics

Item	Set	Add	Sub	Dis	Int	Rot	Com	*p*	$r_{i(t-i)}$	*b*	*a*
1	1	X						.95	.48	-1.28	2.48
2	1		X					.95	.41	-1.49	2.04
3	2			X				.18	.34	1.43	1.14
4	2				X			.90	.34	-2.84	1.04
5	3					X		.91	.31	-2.50	1.09
6	3						X	.95	.23	-3.38	1.00
7	4	X						.98	.34	---	---
8	4		X					.58	.39	-0.01	1.17
9	5			X				.88	.50	-1.36	2.09
10	5				X			.81	.42	-1.24	1.32
11	6					X		.96	.25	-3.25	1.13
12	6						X	.96	.18	-4.08	0.84
13	7	X						.77	.34	-1.05	1.10
14	7		X					.89	.41	-1.39	1.65
15	8			X				.74	.54	-0.36	1.64
16	8				X			.73	.61	-0.19	2.00
17	9					X		.86	.35	-2.17	1.29
18	9						X	.73	.01	-8.98	0.11
19	10	X						.78	.31	-0.55	1.29
20	10		X					.94	.50	-0.79	3.16
21	1	X	X					.84	.47	-0.73	1.74
22	1	X		X				.64	.48	0.13	1.90
23	1	X			X			.82	.46	-0.64	1.70
24	1	X				X		.85	.41	-0.96	1.43
25	1	X					X	.84	.28	-1.28	1.02
26	2		X	X				.40	.53	0.25	1.39
27	2		X		X			.68	.42	-1.26	0.89
28	2		X			X		.72	.57	-1.14	1.54
29	2		X				X	.65	.46	-0.94	1.08
30	2			X	X			.48	.60	-0.10	1.63
31	3			X		X		.46	.50	0.37	1.28
32	3			X			X	.57	.63	-0.05	1.69
33	3				X	X		.45	.56	0.38	1.51
34	3				X		X	.21	.39	1.44	1.46
35	3					X	X	.74	.27	-1.70	0.62
36	4	X	X					.80	.52	-0.71	1.80
37	4	X		X				.71	.50	-0.37	1.63
38	4	X			X			.74	.46	-0.55	1.53
39	4	X				X		.75	.51	-0.52	1.68
40	4	X					X	.77	.31	-1.08	0.99
41	5		X	X				.33	.41	0.89	1.49
42	5		X		X			.50	.59	0.28	2.12
43	5		X			X		.60	.29	-0.39	0.72
44	5		X				X	.59	.36	-0.24	0.90
45	5			X	X			.44	.64	0.45	2.97
46	6			X		X		.74	.40	-1.14	0.98

Item	Set	Add	Sub	Dis	Int	Rot	Com	*p*	$r_{i(t-i)}$	*b*	*a*
47	6			X			X	.57	.48	-0.10	1.27
48	6				X	X		.38	.32	0.84	0.83
49	6				X		X	.66	.37	-0.70	0.91
50	6					X	X	.82	.55	-1.13	1.76
51	7	X	X					.72	.46	-0.53	1.55
52	7	X		X				.66	.51	-0.26	1.79
53	7	X			X			.49	.47	0.26	1.63
54	7	X				X		.86	.40	-1.21	1.69
55	7	X					X	.68	.46	-0.39	1.58
56	8		X	X				.72	.60	-0.16	1.95
57	8		X		X			.63	.61	0.20	2.08
58	8		X			X		.58	.48	0.26	1.44
59	8		X				X	.71	.42	-0.41	1.13
60	8			X	X			.70	.70	0.04	2.76
61	9			X		X		.14	.41	1.37	1.62
62	9			X			X	.29	.53	0.60	1.77
63	9				X	X		.28	.46	0.79	1.28
64	9				X		X	.35	.51	0.45	1.36
65	9					X	X	.87	.34	-2.25	1.23
66	10	X	X					.84	.39	-0.58	1.70
67	10	X		X				.90	.44	-0.70	2.21
68	10	X			X			.80	.38	-0.41	1.74
69	10	X				X		.78	.24	-0.79	1.01
70	10	X					X	.88	.36	-0.80	1.78
71	1	X	X	X				.73	.40	-0.35	1.33
72	1	X	X		X			.61	.33	0.04	1.05
73	1	X	X			X		.74	.42	-0.37	1.39
74	1	X	X				X	.91	.47	-1.06	1.94
75	1	X		X	X			.61	.46	0.21	1.81
76	1	X		X		X		.65	.42	0.02	1.44
77	1	X		X			X	.69	.39	-0.20	1.34
78	1	X			X	X		.78	.56	-0.24	2.41
79	2	X			X		X	.41	.60	0.17	1.84
80	2	X				X	X	.71	.52	-1.12	1.46
81	2		X	X	X			.57	.71	-0.37	2.24
82	2		X	X		X		.65	.65	-0.72	1.94
83	2		X	X			X	.56	.56	-0.42	1.44
84	2		X		X	X		.54	.60	-0.32	1.62
85	2		X		X		X	.63	.72	-0.56	2.56
86	2		X			X	X	.56	.57	-0.39	1.49
87	3			X	X	X		.47	.64	0.33	2.05
88	3			X	X		X	.33	.48	0.89	1.36
89	3				X	X	X	.58	.65	-0.08	2.00
90	3	X	X	X				.47	.57	0.29	1.55
91	3	X	X		X			.70	.74	-0.45	3.02
92	3	X	X			X		.62	.49	-0.38	1.23
93	3	X	X				X	.73	.65	-0.66	2.28
94	3	X		X	X			.56	.62	-0.02	1.91

**Item**	**Set**	**Add**	**Sub**	**Dis**	**Int**	**Rot**	**Com**	p	$r_{i(t-i)}$	b	a
95	4	X		X		X		.49	.46	0.36	1.66
96	4	X		X			X	.63	.51	-0.05	1.83
97	4	X			X	X		.61	.51	0.04	1.94
98	4	X			X		X	.68	.49	-0.22	1.75
99	4	X				X	X	.71	.42	-0.43	1.48
100	4		X	X	X			.62	.56	0.03	2.17
101	4		X	X		X		.68	.61	-0.11	2.55
102	4		X	X			X	.68	.54	-0.14	2.16
103	5		X		X	X		.52	.55	0.21	1.70
104	5		X		X		X	.46	.52	0.41	1.66
105	5		X			X	X	.76	.49	-0.83	1.50
106	5			X	X	X		.71	.64	-0.41	2.26
107	5			X	X		X	.69	.62	-0.36	2.07
108	5				X	X	X	.69	.62	-0.38	2.04
109	5	X	X	X				.71	.68	-0.38	2.65
110	5	X	X		X			.63	.72	-0.07	3.09
111	6	X	X			X		.53	.53	0.13	1.63
112	6	X	X				X	.82	.57	-1.07	1.87
113	6	X		X	X			.51	.56	0.21	1.94
114	6	X		X		X		.57	.46	-0.10	1.20
115	6	X		X			X	.71	.64	-0.46	2.24
116	6	X			X	X		.68	.63	-0.35	2.12
117	6	X			X		X	.73	.64	-0.55	2.19
118	6	X				X	X	.73	.60	-0.58	1.98
119	7		X	X	X			.49	.50	0.28	1.93
120	7		X	X		X		.55	.53	0.12	1.93
121	7		X	X			X	.66	.59	-0.16	2.37
122	7		X		X	X		.71	.52	-0.38	1.94
123	7		X		X		X	.76	.63	-0.42	2.92
124	7		X			X	X	.75	.64	-0.39	2.83
125	7			X	X	X		.52	.59	0.23	2.72
126	7			X	X		X	.77	.66	-0.43	3.30
127	8				X	X	X	.48	.54	0.65	1.98
128	8	X	X	X				.70	.60	-0.11	1.87
129	8	X	X		X			.80	.66	-0.41	2.49
130	8	X	X			X		.73	.64	-0.15	2.18
131	8	X	X				X	.67	.50	-0.09	1.44
132	8	X		X	X			.66	.52	-0.05	1.48
133	8	X		X		X		.69	.64	0.00	2.11
134	8	X		X			X	.71	.61	-0.11	1.98
135	9	X			X	X		.49	.59	-0.13	1.68
136	9	X			X		X	.50	.69	-0.14	2.37
137	9	X				X	X	.65	.56	-0.77	1.65
138	9		X	X	X			.51	.66	-0.20	2.20
139	9		X	X		X		.60	.70	-0.48	2.63
140	9		X	X			X	.37	.48	0.35	1.27
141	9		X		X	X		.60	.73	-0.45	2.84
142	9		X		X		X	.52	.70	-0.20	2.50

Item	Set	Add	Sub	Dis	Int	Rot	Com	*p*	$r_{i(t-i)}$	*b*	*a*
143	10		X			X	X	.71	.37	-0.12	1.56
144	10			X	X	X		.68	.54	0.21	3.04
145	10			X	X		X	.69	.52	0.16	2.86
146	10				X	X	X	.80	.59	-0.04	3.63
147	10	X	X	X				.69	.34	-0.08	1.41
148	10	X	X		X			.20	.19	1.65	1.36
149	10	X	X			X		.85	.44	-0.45	2.16
150	10	X	X				X	.65	.33	0.05	1.40
151	1	X	X	X	X			.53	.40	0.43	1.51
152	1	X	X	X		X		.60	.47	0.27	1.93
153	1	X	X	X			X	.64	.39	0.03	1.44
154	1	X	X		X	X		.67	.64	0.22	4.05
155	1	X	X		X		X	.64	.51	0.21	2.44
156	2	X	X			X	X	.49	.64	-0.12	1.85
157	2	X		X	X	X		.43	.62	0.10	2.01
158	2	X		X	X		X	.48	.75	-0.03	3.78
159	2	X			X	X	X	.50	.63	-0.12	2.19
160	2		X	X	X	X		.39	.63	0.25	2.94
161	3		X	X	X		X	.48	.57	0.26	1.57
162	3		X	X		X	X	.42	.56	0.48	1.74
163	3		X		X	X	X	.53	.73	0.17	3.35
164	3			X	X	X	X	.34	.45	0.87	1.28
165	3	X	X	X	X			.41	.54	0.53	1.55
166	4	X	X	X		X		.35	.41	0.76	2.01
167	4	X	X	X			X	.57	.57	0.21	2.67
168	4	X	X		X	X		.62	.59	0.09	2.95
169	4	X	X		X		X	.66	.66	0.05	3.86
170	4	X	X			X	X	.62	.70	0.18	5.16
171	5	X		X	X	X		.38	.53	0.63	2.06
172	5	X		X	X		X	.51	.65	0.26	2.68
173	5	X			X	X	X	.59	.71	0.05	3.13
174	5		X	X	X	X		.53	.70	0.22	3.12
175	5		X	X	X		X	.46	.66	0.42	3.17
176	6		X	X		X	X	.58	.61	0.01	2.22
177	6		X		X	X	X	.55	.58	0.07	1.97
178	6			X	X	X	X	.55	.66	0.15	3.03
179	6	X	X	X	X			.53	.62	0.17	2.65
180	6	X	X	X		X		.53	.62	0.18	2.44
181	7	X	X	X			X	.56	.48	0.06	1.77
182	7	X	X		X	X		.46	.54	0.37	2.52
183	7	X	X		X		X	.66	.66	-0.09	3.24
184	7	X	X			X	X	.58	.51	0.04	2.02
185	7	X		X	X	X		.42	.58	0.46	3.68
186	8	X		X	X		X	.63	.57	0.16	1.77
187	8	X			X	X	X	.63	.60	0.20	2.12
188	8		X	X	X	X		.63	.65	0.24	2.49
189	8		X	X	X		X	.66	.72	0.20	3.35
190	8		X	X		X	X	.62	.71	0.35	3.62

Item	Set	Add	Sub	Dis	Int	Rot	Com	*p*	$r_{i(t-i)}$	*b*	*a*
191	9		X		X	X	X	.44	.72	0.05	3.08
192	9			X	X	X	X	.44	.69	0.04	2.85
193	9	X	X	X	X			.38	.64	0.24	2.41
194	9	X	X	X		X		.29	.52	0.60	1.78
195	9	X	X	X			X	.36	.65	0.31	2.69
196	10	X	X		X	X		.70	.56	0.17	3.30
197	10	X	X		X		X	.68	.50	0.19	2.86
198	10	X	X			X	X	.42	.24	0.88	1.08
199	10	X		X	X	X		.74	.63	0.18	5.08
200	10	X		X	X		X	.62	.51	0.35	3.46
201	1	X	X	X	X	X		.31	.35	1.11	1.74
202	1	X	X	X	X		X	.55	.50	0.44	2.58
203	2	X	X	X		X	X	.38	.66	0.27	3.74
204	2	X	X		X	X	X	.36	.64	0.31	3.82
205	3	X		X	X	X	X	.46	.70	0.40	3.40
206	3		X	X	X	X	X	.37	.60	0.65	2.81
207	4	X	X	X	X	X		.45	.56	0.49	4.00
208	4	X	X	X	X		X	.44	.55	0.50	3.63
209	5	X	X	X		X	X	.31	.53	0.80	2.60
210	5	X	X		X	X	X	.42	.63	0.53	3.43
211	6	X		X	X	X	X	.48	.61	0.33	2.91
212	6		X	X	X	X	X	.45	.59	0.39	3.02
213	7	X	X	X	X	X		.23	.43	0.91	3.18
214	7	X	X	X	X		X	.39	.59	0.52	4.48
215	8	X	X	X		X	X	.34	.42	1.09	1.89
216	8	X	X		X	X	X	.47	.57	0.69	2.49
217	9	X		X	X	X	X	.29	.59	0.50	2.97
218	9		X	X	X	X	X	.29	.62	0.50	3.63
219	10	X	X	X	X	X		.50	.50	0.58	4.04
220	10	X	X	X	X		X	.06	.13	2.41	1.63

Notes: Add, addition; Sub, subtraction, Dis, disjunctive union; Int, intersection; Rot, rotation; Com, completeness; *p*, item solution probability, $r_{i(t-i)}$, part-whole correlation; *b*, item parameter; *a*, item discrimination parameter; ---, not estimated due to model misfit; In the rows Add to Com an X marks that this rule was used for the construction of the item.
